# Asymptomatic infections with highly polymorphic Chlamydia suis are ubiquitous in pigs

**DOI:** 10.1186/s12917-017-1295-x

**Published:** 2017-12-01

**Authors:** Min Li, Martina Jelocnik, Feng Yang, Jianseng Gong, Bernhard Kaltenboeck, Adam Polkinghorne, Zhixin Feng, Yvonne Pannekoek, Nicole Borel, Chunlian Song, Ping Jiang, Jing Li, Jilei Zhang, Yaoyao Wang, Jiawei Wang, Xin Zhou, Chengming Wang

**Affiliations:** 1 Yangzhou University College of Veterinary Medicine, Yangzhou, Jiangsu People’s Republic of China; 2 Centre for Animal Health Innovation, Faculty of Science, Health, Education & Engineering, University of the Sunshine Coast, QLD, Maroochydore, Australia; 3 Poultry Institute, Chinese Academy of Agricultural Sciences, Yangzhou, Jiangsu China; 4 College of Veterinary Medicine, Auburn University, Auburn, Alabama USA; 5 Institute of Veterinary Medicine, Jiangsu Academy of Agricultural Sciences; Key Laboratory of Veterinary Biological Engineering and Technology, Ministry of Agriculture, National Center for Engineering Research of Veterinary Bio-Products, Nanjing, China; 6 Academic Medical Center, University of Amsterdam, Amsterdam, The Netherlands; 7 Institute for Veterinary Pathology, Vetsuisse Faculty, University of Zurich, Zurich, Switzerland; 8 Yunnan Agricultural University College of Animal Science & Technology, Kunming, Yunnan China; 9 Key Laboratory of Animal Diseases Diagnostic and Immunology, Ministry of Agriculture, College of Veterinary Medicine, Nanjing Agricultural University, Nanjing, China

**Keywords:** *Chlamydia suis*, Pig, FRET-PCR, *ompA*, MLST, Tanglegram

## Abstract

**Background:**

*Chlamydia suis* is an important, globally distributed, highly prevalent and diverse obligate intracellular pathogen infecting pigs. To investigate the prevalence and genetic diversity of *C. suis* in China, 2,137 nasal, conjunctival, and rectal swabs as well as whole blood and lung samples of pigs were collected in 19 regions from ten provinces of China in this study.

**Results:**

We report an overall positivity of 62.4% (1,334/2,137) of *C. suis* following screening by *Chlamydia* spp. 23S rRNA-based FRET-PCR and high-resolution melting curve analysis and confirmatory sequencing. For *C. suis*-positive samples, 33.3 % of whole blood and 62.5% of rectal swabs were found to be positive for the *C. suis tetR*(C) gene, while 13.3% of whole blood and 87.0% of rectal swabs were positive for the *C. suis tet*(C) gene. Phylogenetic comparison of partial *C. suis ompA* gene sequences revealed significant genetic diversity in the *C. suis* strains. This genetic diversity was confirmed by *C. suis*-specific multilocus sequence typing (MLST), which identified 26 novel sequence types among 27 examined strains. Tanglegrams based on MLST and *ompA* sequences provided evidence of *C. suis* recombination amongst the strains analyzed.

**Conclusions:**

Genetically highly diverse *C. suis* strains are exceedingly prevalent in pigs. As it stands, the potential pathogenic effect of *C. suis* on pig health and production of *C. suis* remains unclear and will be the subject of further investigations. Further study is also required to address the transmission of *C. suis* between pigs and the risk of 'spill-over' and 'spill-back' of infections to wild animals and humans.

**Electronic supplementary material:**

The online version of this article (10.1186/s12917-017-1295-x) contains supplementary material, which is available to authorized users.

## Background

Obligate intracellular bacteria of the genus *Chlamydia* are the etiological agents of chlamydiosis in wild and domestic birds, mammals and humans [1–4]. Eleven recognized *Chlamydia* spp. (*C. trachomatis*, *C. suis*, *C. muridarum*, *C. pneumoniae*, *C. abortus*, *C. caviae*, *C. felis*, *C. pecorum*, *C. psittaci*, *C. avium* and *C. gallinacea*) are included in the single genus *Chlamydia* under the family *Chlamydiaceae* currently contains [5].

In pigs, four chlamydial species (*C. pecorum*, *C. abortus*, *C. psittaci* and *C. suis*) are regularly detected [6]. Of these, *C. abortus* and especially *C. suis* are the main species and most common involved in porcine chlamydial infections, with mixed infections occurring regularly [7–10] and only a limited number of reports finding a single species (*C. suis*) infecting cohorts of pig [10, 11].

In pigs, *C. suis* is mainly associated with subclinical infections, however it has also been found in association with respiratory disease, diarrhea, conjunctivitis and reproductive failure [3, 6]. Beyond pigs, DNA from this organism has also been detected in sheep, cattle, horses, cats, kestrels, and frogs [12–14]. Whether this PCR-based evidence represents the detection of an active infection versus exposure is, however. unknown [12–14].

Zoonotic potential of *C. suis* was suggested in the previous study, showing a single or mixed infection with *C. trachomatis* [15]. In addition, conjunctival swabs of employees in a Belgian pig slaughterhouse was identified to carry *C. suis* DNA [3] which was also found in the and pharyngeal and rectal swabs of Belgian pig farmers [10].

Consequently, pig pathogens may not only affect the pig production, but also potentially play a role in public health.

For decades, tetracyclines, as broad-spectrum antibiotics, have been extensively used in the pig industry for both prophylactic and therapeutic treatment. Interestingly, *C. suis* is the only chlamydial species to have naturally acquired genes encoding for antibiotic resistance. Over the past years, there have been accumulating reports on the occurrence of tetracycline-resistant *C. suis* strains in America, Europe and Asia [16–20]. This resistance pattern is associated with *tet*(C) islands. No standard antibiotic treatment of pigs (amoxicillin, chlortetracycline, chlortetracycline plus sulfadimidine, tylosin, trimethoprime plus sulfadimidin plus sulfathiazole) appear to be able to clear chlamydial infections at the herd level, despite individual pigs becoming negative [21]. Moreover, clear evidence of selective pressure was obvious as tetracycline treatment resulted in a higher number of tetracycline-resistant *C. suis* isolates [22]. Highlighting the additional concerns over the presence of this antibiotic-resistance genes in *C. suis*, transfer of tetracycline resistance from *C. suis* to *C. trachomatis* and *C. muridarum*, and between *C. suis* and *C. trachomatis* strains has been demonstrated *in vitro* [23].

Currently, there is no standardized typing scheme to evaluate the genetic diversity of *C. suis*. However, molecular typing schemes for other *Chlamydiaceae* are primarily based on characterizing epitopes in the major chlamydial outer membrane protein. For the closely related *C. trachomatis*, variants of this protein are subjected to selection and isolates of the same serovar may not be closely related [24, 25]. A recent study has revealed that *C. suis* had the highest whole genome recombination rate of *Chlamydia* species studied to date by whole genome sequencing [26] with *ompA,* as one of 77 *C. suis* core genes, showing significant evidence for intragenic recombination. As an alternative to *ompA*-based typing schemes, multilocus sequence typing (MLST) schemes that typically target fragments of five to seven housekeeping (HK) genes under neutral selection are widely used for determining bacterial population structures and barcoding isolates [27].

We have recently shown that, in China, a range of important livestock species, including poultry [28] and cattle [29], are infected with different endemic and epidemic strains of *Chlamydia*. Pig production is a major agricultural industry in China, however, little is known about the diversity of chlamydial infections that might impact on the health of pigs in this country. Therefore, the present study was undertaken to investigate the prevalence of *Chlamydia* spp. in pigs from China, with a particular emphasis on *C. suis* infections. Given the potential importance of tetracycline resistance for the latter species, we also investigated the presence of tetracycline resistance genes in these strains. To analyze diversity, phylogenetic and cluster analyses of *C. suis* was performed using both *ompA* and a *C. suis*-specific MLST typing scheme designed for the purpose of this study.

## Methods

### Ethics statement

Protocols for the collection of samples in this study were reviewed and approved by the Institutional Animal Care and Use Committee of Yangzhou University College of Veterinary Medicine (YZU-CVM#2015-057). The written permission was obtained from the owners of the pigs used in this study.

### Sample collection

#### Nasal, rectal swabs, whole blood and lung samples

In this study, a total of 2,137 clinical samples (lung, whole blood, rectal swabs, nasal swabs, conjunctival swabs) were collected from pigs in the farms of 19 regions of 10 provinces of China between 2015 and 2016 (Table 1). A part of samples were collected from pigs in 9 regions of Jiangsu province: whole blood samples (n=308) and rectal swabs (n=55) were collected in Yangzhou City, whole blood samples (n=33) were from Nantong city, nasal swabs were from Nanjing city (n=180), Huaian city (n=70), Suqian city (n=41), Taizhou city (n=49), Wuxi city (n=36), Yancheng city (n=37), Changzhou city (n=23). In addition, nasal swabs (n=68) and rectal swabs (n=68) were collected from swine farms in Kunming city in Yunnan province; nasal swabs were from Shanghai municipality (n=52), Xihua City in Henan province (n=63), Changchun City in Jilin province (n=63), Jiangmen City in Guangdong province (n=40), Haerbin City in Heilongjiang province (n=60) and Jining City in Shandong province (n=60), respectively. Nasal swabs were also collected from Specific Pathogen Free (SPF) pigs (n=108) in two experimental animal companies in Nanjing City in Jiangsu province. Lung tissue samples (n=23) from an abattoir in Nanping city in Fujian province were also collected.

**Table 1:** Prevalence and copy numbers of *C. suis* of pigs from provinces of China

Province	City	Sample type	Age	Positivity	Copy /ml or swab, log10
Fujian	Nanping	Lung	NA	26.1% (6/23)	3.72 (40mg)
Jiangsu	Yangzhou	Whole blood	Finisher pig	33.4 % (103/308)	2.79
		Rectal swab	Finisher pig	98.2%(54/55)	4.50
	Nantong	Whole blood	Suckling pig	0.0% (0/11)	0.00
			Weaned pig	20.0% (2/10)	3.17
			Sow	8.3% (1/12)	2.85
	Nanjing	Nasal swab	Weaned pig*	86.1% (93/108)	3.65
			35d	95.0%(57/60)	2.61
			49d	76.7%(46/60)	1.85
			63d	91.7%(55/60)	4.19
	Huaian	Nasal swab	Suckling pig	40.9% (9/22)	2.16
			Weaned pig	18.8% (3/16)	1.99
			Finisher pig	58.3% (7/12)	2.11
			Sow	15.0% (3/20)	1.99
	Suqian	Nasal swab	Suckling pig	40.0% (6/15)	2.68
			Weaned pig	38.9% (7/17)	3.07
			Sow	12.5% (1/9)	2.20
	Taizhou	Nasal swab	Suckling pig	69.2% (18/26)	2.25
			Weaned pig	69.2 (9/13)	2.91
			Finisher pig	100.0% (5/5)	3.67
			Sow	0.0% (0/5)	0.00
	Wuxi	Nasal swab	Suckling pig	17.6% (3/17)	1.99
			Weaned pig	100.0% (5/5)	3.16
			Finisher pig	100.0% (5/5)	3.13
			Sow	0.0% (0/9)	0.00
	Yancheng	Nasal swab	Suckling pig	43.8% (7/16)	2.13
			Weaned pig	87.5% (7/8)	2.95
			Finisher pig	100.0% (3/3)	2.89
			Sow	30.0% (3/10)	2.31
	Changzhou	Nasal swab	Suckling pig	100% (7/7)	3.48
			Weaned pig	100.0% (16/16)	3.81
Shanghai	Shanghai	Nasal swab	Suckling pig	66.7% (12/18)	2.60
			Weaned pig	57.1% (8/14)	2.12
			Finisher pig	100.0% (10/10)	3.89
			Sow	20.0% (2/10)	3.00
Yunnan	Kunming	Nasal swab	Suckling pig	92.3% (12/13)	2.91
			Weaned pig	100.0% (9/9)	3.80
			Finisher pig	86.7% (13/15)	2.25
			NA	90.3% (28/31)	3.12
		Rectal swab	Suckling pig	84.6% (11/13)	3.03
			Weaned pig	100.0% (9/9)	4.14
			Finisher pig	66.7% (10/15)	2.58
			NA	93.6% (29/31)	4.54
Zhejiang	Shaoxing	Nasal swab	Suckling pig	89.0% (89/100)	3.55
			Weaned pig	95.0% (95/100)	4.26
			Finisher pig	82.0% (41/50)	3.27
			Sow	46.0% (23/50)	2.97
	Shaoxing	Nasal swab	Sow	32.0%(16/50)	2.74
		Conjunctival swab	Sow	26.0% (13/50)	2.56
		Rectal swab	Sow	20.0% (10/50)	2.89
		Whole blood	Sow	0.0% (0/50)	0.00
	Ningbo	Nasal swab	Weaned pig	100.0% (50/50)	4.86
		Conjunctival swab		100.0% (50/50)	4.87
		Rectal swab		100.0% (50/50)	5.83
		Whole blood		16.0% (8/50)	3.50
Henan	Xihua	Nasal swab	Sow	28.1%(9/32)	3.13
			Weaned pig	96.8%(30/31)	3.42
Jilin	Changchun	Nasal swab	Weaned pig	81.1%(53/63)	3.94
Guangdong	Jiangmen	Nasal swab	Weaned pig	100.0%(20/20)	4.86
			Sow	90.0%(18/20)	3.68
Heilongjiang	Haerbin	Nasal swab	Suckling pig	71.7%(43/60)	2.07
Shandong	Jining	Nasal swab	Suckling pig	53.3%(16/30)	3.68
			Sow	6.7%(2/30)	2.84

*represents SPF weaned pig from two experimental animal companies. NA: not applicable.

Nasal swabs, conjunctival and rectal swabs were collected into sterile Eppendorf tubes (Eppendorf, Shanghai, China) containing 400 μl DNA/RNA stabilization buffer (Roche Molecular Biochemicals, Indianapolis, IN, USA). Blood samples were collected into EDTA tubes (Becton, Dickinson and Company, Franklin Lakes, NJ, USA) and transported and stored at room temperature.

#### Nasal swabs from pigs of different ages

To compare and analyze differences in positivity and infectious load of *C. suis* among suckling pigs (age before 25 days old), weaned pigs (age after 25 d), finisher pigs (age after 100 d) and sows (age after 210 d), nasal swabs were collected from animals in each age group from Shaoxing city in Zhejiang Province: suckling pigs (n=100), weaned pigs (n=100), finisher pigs (n=50) and sows (n=50).

#### Nasal, conjunctival, rectal swabs and whole blood samples from pigs on two pig farms in Zhejiang province

To compare and analyze differences in the shedding of *C. suis* from different anatomical sites of animals, multiple samples (nasal, conjunctival, rectal swabs and whole blood) were collected randomly from 50 sows in a pig farm in Shaoxing of Zhejiang province and 50 weaned pigs in Ningbo of Zhejiang province. For the former, 50 sows in two blocks were randomly chosen out of 160 sows on pig farms in Shaoxing. For the latter, 50 weaned pigs in 10 blocks were chosen out of 200 weaned pigs on pig farms in Ningbo for this study. Whole blood samples (around 2 ml) were collected in EDTA tubes, nasal, conjunctival, rectal swabs were collected as above mentioned.

### DNA extraction from swabs, whole blood and lung samples

The High-Pure PCR Template Preparation Kit (Roche Molecular Biochemicals, Indianapolis, IN, USA) was used to extract total nucleic acids from nasal, conjunctival, rectal swabs and whole blood and lungs from pigs, according to the manufacturer's instructions and described before [30]. The extracted DNA was eluted in 200 μl elution buffer. In this study, swabs obtained weekly from research laboratory were processed for *Chlamydia* qPCR to verify free of carry-over contamination occurred. Furthermore, diethylpyrocarbonate (DEPC)-treated ddH_2_O served as a negative control to confirm that contamination between samples did not occur during the DNA extraction.

### *Chlamydia* FRET-PCR

The FRET-PCR was performed in a LightCycler 480-II real-time PCR platform., and the protocol of the PCR in this study followed what described [28, 31]. This 23S rRNA-based FRET-PCR was able to detect all 11 *Chlamydia* species and had a detection limit of single copies/reaction. The PCR products were further verified by electrophoresis followed by DNA sequencing (BGI, Shanghai, China).

### *Tet*(C) and *tetR*(C) PCR

Whole blood samples of both *C. suis*-positive (n=45) and *C. suis*-negative (n=40) from Yangzhou in Jiangsu province and rectal swabs of both *C. suis*-positive (n=54, 45 from Kunming in Yunnan province and 9 from Shaoxing in Zhejiang province) and *C. suis*-negative (n=53, 38 from Kunming in Yunnan province and 15 from Shaoxing in Zhejiang province) were examined for presence of the tetracycline resistance gene, *tet*(C), and the tetracycline repressor gene, *tetR*(C), by PCR as previously described [17].

### *C. suis*-specific *ompA*-PCRs

To investigate the polymorphisms in the *C. suis ompA* gene, a set of primers were designed using Vector NTI to amplify the *ompA* VD 1-2 (amplicon size: 491bp) to interrogate 108 *C. suis* -positive samples (44 whole blood, 3 lungs samples, 12 rectal swabs, 1 conjunctival swabs, 48 nasal swabs). Twenty μl PCRs were prepared containing 10.0 μl DNA template, 0.2μl forward primer (100 μM), 0.2μl reverse primer (100 μM,), 4.0 μl 5 x PCR buffer, 0.4 μl 10 μM dNTP, 0.3 μl 5 U/μl *Taq* DNA polymerase and 4.9 μl Ultrapure H_2_O. In addition, two sets of primers were designed to specifically amplify the *ompA* gene of *C. abortus* and *C. pecorum* (Table 2). Along with a set of specific primers for *C. psittaci* [29], these assays were used to confirm the detection of each species’ DNA from the *Chlamydia* FRET-qPCR. PCR amplification was performed in a LightCycler 480-II real-time PCR platform using a high-stringency 18-cycle step-down temperature protocol: 6 x 1 sec @ 95^o^C, 12 sec @64^o^C, 8 sec @ 72^o^C; 9 x 1 sec @ 95^o^C, 12 sec @ 62^o^C, 8 sec @ 72^o^C; 3 x 1 sec @ 95^o^C, 12 sec @ 60^o^C, 8 sec @ 72^o^C; followed by 30 low-stringency cycles: 30 x 1 sec @ 95^o^C, 12 sec @ 55^o^C, 30 sec @ 67^o^C, and 10 sec @ 72^o^C. The PCR products were further verified, purified and sequenced as mentioned above.

**Table 2:** *ompA* and MLST primers used in this study

*Chlamydia*	Target	Primer/probe	Sequence (5'-3')	Amplicon Size (bp)	MLST fragment (bp)
*C. suis*	*Cs*_*ompA* VD 1-2	UP	TTGAACATTTGGGATCGTTTTGA	491	_
		DN	CCAATGTAAGGAGTGAACATATTTAATCTG		
	*gatA*	UP	TAAAAGTGCTTTAGAATTAAGAGATGCTGT	539	425
		DN	AGATGCTGGCTGACGAATCGA		
	*oppA*_3	UP	AGATATCAGTGGGAATCTGCTTGC	674	468
		DN	TAAGGATTTTTTTCCAATTTAAGCCAT		
	*hflX*	UP	CTCTCCCTCTCAACAACGGAACTT	616	435
		DN	TTCAATAACATGCAGCAAAATATCCTC		
	*gidA*	UP	TTTGGGAGTTTCTACGAAGGAAGG	570	474
		DN	ATAATTTCATATTGTACATCGAAAGGCAT		
	*enoA*	UP	TCTCGGGGTCTCTTTAGCATTAGC	590	381
		DN	CTCCAACGAGTTGAATACGATCTCC		
	*hemN*	UP	GAAGAGCTTGCTATTGAATTTGATCC	608	432
		DN	CGTTTTGTAGATAGATTCCTCGAATGA		
	*fumC*	UP	CTCTTATGGGAAAGAATTGATGCCT	641	465
		DN	TACTTTCTCTACAAAACCTTCAGGAACATT		
*C. abortus*	Cab_*ompA*	UP	TACAAGCCTTGCCTGTAGGGAAC	360	_
		DN	CAGAAAATATCAAAGCGATCCCAG		
*C. pecorum*	Cpe*_ompA*	UP	ATGAAAAAACTCTTAAAATCGGCGT	420	_
		DN	CAGAAAATATCAAAGCGATCCCAG		

### *C. suis*-specific MLST analysis

In this study, a *C. suis*-specific MLST typing scheme based on a previously published *Chlamydiales* MLST scheme [32, 33] was developed. The scheme was designed to target the partial fragments of seven *C. suis* HK genes. The selected genes are not adjacent to putative outer membrane, secreted, or hypothetical and are separated widely on the chromosome. In addition, each locus for the selected genes demonstrated a similar degree of nucleotide substitutions to provide consistency [27].

Amplification primers were designed based on the genome sequence of *C. suis* MD56 to amplify fragments of the genes encoding aspartyl/glutamyl-tRNA amidotransferase subunit A (*gatA*) (amplicon length: 539 bp), oligobinding protein (*oppA*) (674 bp), GTP-binding protein (*hflX*) (616bp), tRNA uracil-5-methyl transferase (*gidA*) (570 bp), enolase (*enoA*) (590 bp), Coproporphyrinogen III oxidase (*hemN*) (608bp), and fumarate hydratase class II (*fumC*) (641bp) (Table 2). PCR amplification of the seven HK genes was performed in a LightCycler 480-II real-time PCR platform following the above-mentioned protocol. Each sequence run was performed from a different PCR amplicon and sequence traces were obtained with automated DNA sequencing. Briefly, forward and reverse chromatograms for each sequenced HK gene fragment were aligned and trimmed, and the fragment sequence for that allele was obtained. Sequences for each gene were then aligned in MEGA 6.0 using the ClustalW multiple alignment algorithm and trimmed to appropriate lengths for *Chlamydiales* MLST fragments [32].

After the optimization and development, the *C. suis*-MLST was successfully applied to *C. suis* positive samples from nasal (n=12) and rectal (n=7) swabs, 6 whole blood (n=6) and 1 lung samples (n=1) from 26 pigs. Phylogenetic analyses were performed using DNASp 5.0 [34] and Geneious 9 [35]. DnaSP 5.0 was used to analyze the sequence polymorphisms: by determining the number of synonymous (dS) and non-synonymous (dN) substitutions per site, Jukes-Cantor corrected, the number of polymorphic sites, haplotypes and haplotypesper allele. Allele and sequence type (ST) assignation for 26 *C. suis* strains described in this study and one Italian strain-MD56 were determined and deposited at http://pubmlst.org/chlamydiales/ [36].

### Phylogenetic analysis

Phylogenetic analyses were performed using the 489 bp variable region of the *ompA* gene and the concatenated *C. suis* MLST sequences. For *ompA*, a total of 127 sequences consisting of 50 publicly available sequences obtained from GenBank and 77 sequences from this study were aligned using the ClustalX 1.83. Based on these alignments, the NJ phylogenetic trees were constructed using the Kimura 2-parameter model. The MEGA 6.0. Bootstrap values were calculated by the use of 500 replicates.

A Bayesian phylogenetic tree using an alignment of a total of 40 concatenated MLST sequences from the 26 *C. suis* strains from this study, and 14 additional strains from Switzerland, USA, Italy and Austria. *C. suis* isolates was constructed with MRBAYES [37] with the GTR+G model, as implemented in Geneious 9. Run parameters included four Markov Chain Monte Carlo (MCMC) chains with a million generations, sampled every 1000 generations and with the first 10000 trees were discarded as burn-in. *C. trachomatis* MLST sequences were used as an outgroup to determine the mid-point root of the *C. suis* phylogeny. MLST gene fragments were extracted from publicly available genomes of the strains SWA-2 (GenBank acc. No. NZ_LT821323), 14-23b (NZ_FSSG01000023), 1-28b (NZ_FTQD01000002), 3-25a (NZ_FTQO01000003), 10-26b (NZ_FTQJ01000001), 5-22b (NZ_FTQB01000001), 8-29b (NZ_FTQU01000001), 3-29b (NZ_FTPY01000001- NZ_FTPY01000002), S45 (SRA accession: SRX1868493), R19 (SRX1868490), Rogers130 (SRX1868491), R1 (SRX1868495), R16 (SRX1868494), MD56 (NZ_AYKJ00000000).

Minimum spanning tree and identification of clonal complexes (CCs) using the goeBURST algorithm [38] on the *C. suis* MLST data from this study was performed using the https://online.phyloviz.net [39]. The MLST profiles were clustered into CCs under a user-defined threshold level of identity. In this study, we used relaxed triple locus variant (TLV) level where CCs were defined as groups of sequencing types (STs) which share four out of seven alleles with at least one other ST in the group, while the satellite STs were defined as STs that differ by at least three alleles from all other STs. A 'putative progenitor' in CCs is a ST that has the most single locus variant (SLV) links to other STs.

In order to compare phylogenetic inferences based on MLST and *ompA* alignments, we have constructed a tanglegram from cladograms of Bayesian *C. suis* MLST and *ompA* phylogenetic trees, and a strict consensus tree, computed with Dendroscope 3 [40]. For this analysis, we have also constructed a mid-point rooted Bayesian phylogenetic tree using the 489 bp *ompA* fragment alignment from the same 40 (26 Chinese and 14 global) *C. suis* strains used for MLST-derived phylogeny. The tree parameters included GTR+I model and four Markov Chain Monte Carlo (MCMC) chains with a million generations, sampled every 1000 generations and with the first 10000 trees were discarded as burn-in.

In order to infer recombination break points within each alignment, we used Dual-Brothers recombination detection with default setting (as implemented in Geneious 9). DualBrothers detects recombination based on the dual Multiple Change-Point (MCP) model which finds changes in topology and evolutionary rates across sites in a multiple sequence alignment [41].

### Statistical analysis

The Chi-squared Test was used to compare the positivity of *C. suis* infection in different samples of pigs. The two-tailed Tukey honest significant difference (HSD) test in one-way ANOVA was used to analyze the differences of *C. suis* copy numbers in different samples. Differences at P≤0.05 were considered significant.

## Results

### Prevalence of *Chlamydia* spp. in pigs

*C. suis* was found to be the only chlamydial species in all samples in this study with an overall positivity of 62.4% (1,334/2,137). In addition, 26.1% (6/23) of lung samples from Fujian province tested positive for *C. suis* (Table 1). Notably, a cohort of SPF pigs had 80.0% (52/65) of *C. suis* positivity in pigs less than two months old and 95.4% positivity (41/43) in pigs older than two months (Table 1).

### Prevalence and copy numbers of *C. suis* affected by samples types

**Fig. 1.**
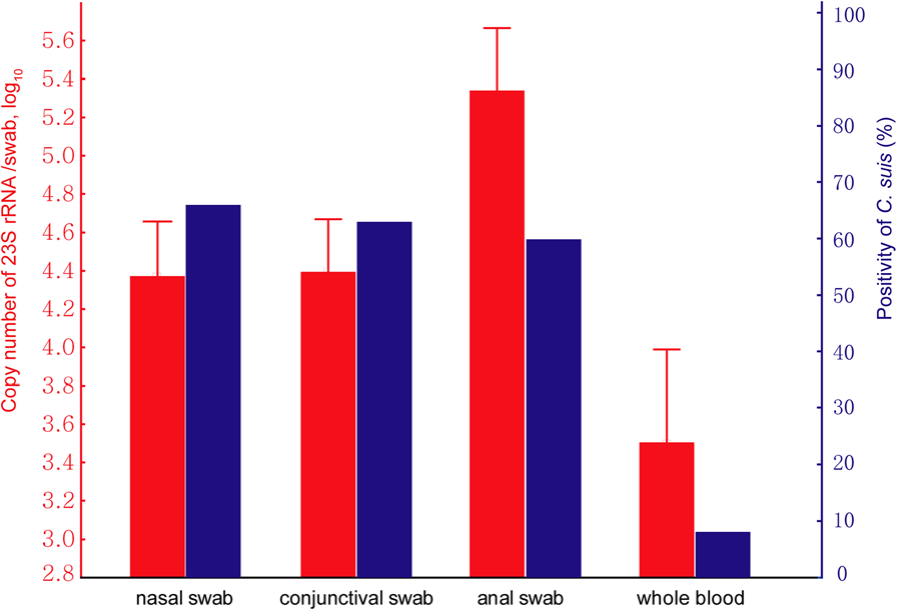
Prevalence and copy numbers of *C. suis* affected by samples type of pigs. Copy number (± SEM) is shown in the left ordinate (in red). Positivity is shown in the right ordinate (in blue). FRET-qPCR were performed to determine the positivity and copy number of *C. suis* from nasal, conjunctival, rectal swabs and whole blood samples of 50 sows and 50 weaned pigs in this study. The positivity of *C. suis* in nasal swabs (66%), conjunctival swabs (63%) and rectal swabs (60%) was significantly higher than whole blood (P<0.01, 8%). The average copy number of *C. suis* was highest in rectal swab (P<0.01, 10^5.34±0.16 [SEM]^ per swab), followed by conjunctival swab (10^4.39±0.14^ per swab) and nasal swab (10^4.37±0.14^ per swab), and whole blood (P<0.05, 10^3.50±0.20^ per ml).

Nasal swabs, conjunctival swabs, rectal swabs and whole blood samples were collected from 100 pigs in Zhejiang province for *C. suis* detection. The prevalence and average bacterial genome numbers of whole blood samples was significantly lower than in other sample types (P<0.05). The *C. suis* positivity was 8.0% (8/100) in whole blood, 66.0% (66/100) in nasal swabs, 63.0% in conjunctival swabs (66/100), and 60.0% in rectal swabs (60/100). In addition, the average bacterial genome numbers of *C. suis* were 10^3.50±0.20 [SEM]^ per ml whole blood), 10^5.34±0.16^ per rectal swab; 10^4.39±0.14^ per conjunctival swab, and 10^4.37±0.14^ per nasal swab (Fig. 1).

### *C. suis* in pigs of different ages

**Fig. 2.**
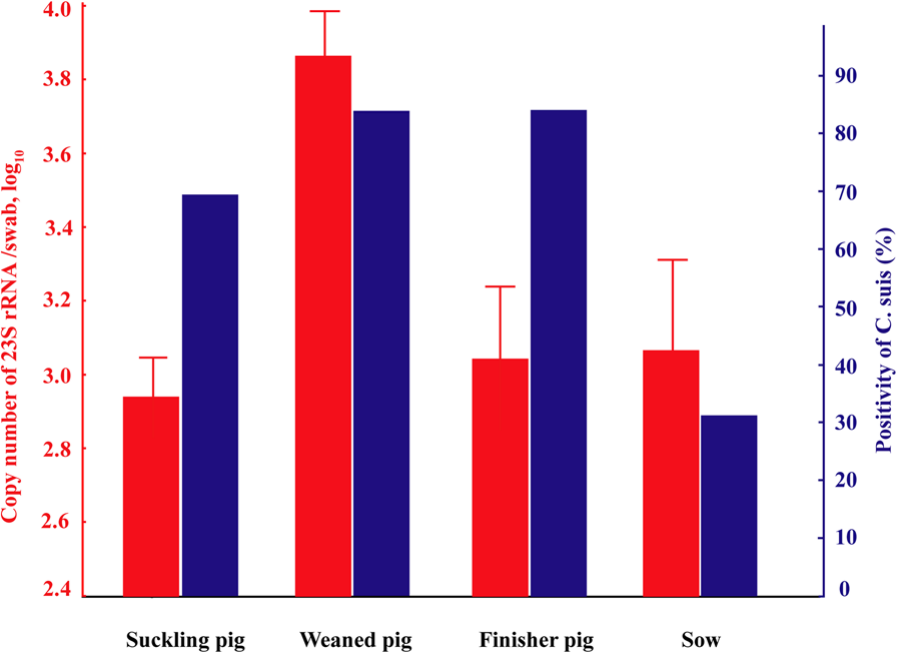
Prevalence and copy numbers of *C. suis* affected by ages of pigs. Copy number (± SEM) is shown in the left ordinate (in red). Positive rate is shown in the right ordinate (in blue). FRET-qPCR were performed to determine the positivity and copy number of *C. suis* from nasal swabs of suckling pigs (n=324), weaned pigs (n=312), finisher pigs (n=100) and sows (n=195) in this study. The positivity of *C. suis* was the lowest in sow (P<0.01, 31.3%), followed by finisher pigs (84.0%) and weaned pigs (84.0%), and suckling pigs (P<0.05, 69.4%). The average copy number of *C. suis* was significantly higher in weaned pigs (P<0.01, 10^3.88±0.06 [SEM]^ per swab) than sows (10^3.11±0.09^ per swab), finisher pigs (10^3.09±0.07^ per swab) and suckling pigs (10^2.94±0.05^ per swab).

*C. suis* positivity in nasal swabs was found to be similar in finisher pigs (84.0%; 84/100) and weaned pigs (84.0%; 262/312), which were significantly higher than in suckling pigs (69.4%, 225/324; P<0.01) and sows (31.3%, 61/195; P<0.01). Interestingly, the average copy number of *C. suis* was significantly higher in weaned pigs (P<0.01, 10^3.88±0.06 [SEM]^ per swab) than in sows (10^3.11±0.09^ per swab), finisher pigs (10^3.09±0.07^ per swab) and suckling pigs (10^2.94±0.05^ per swab) (Fig. 2).

### *Tet*(C) and *tetR*(C) genes

For *C. suis*-positive samples, 6/45 (13.3%) of whole blood samples from Yangzhou in Jiangsu, 39/45 (86.7%) of rectal swabs from Kunming in Yunnan and 8/9 (88.9%) of rectal swabs from Shaoxing in Zhejiang province contained the *tet*(C) gene while 15/45 (33.3%) of whole blood samples and 5/8 (62.5%) of rectal swabs samples were found to be positive for the *tetR*(C) gene while 6 of 45 (13.3%) whole blood samples and 47 of 54 (87.0%) rectal swabs samples contained the *tet*(C) gene. We also tested the presence of *tet*(C) genes in *C. suis*-negative samples in this study. For *C. suis*-negative samples, no (0/40; 0.0%) whole blood samples from Yangzhou in Jiangsu and 19/38 (50%) of 53 (54.7%) rectal swabs from Kunming in Yunnan and 10/15 (66.7%) of rectal swabs from Shaoxing in Zhejiang province contained the *tet*(C) gene. Both *C. suis tet*(C) and *tetR*(C) were detected in samples from pigs in 3 provinces sampled in this study. In a word, the *tet*(C) positivity in *C. suis*-positive samples was significantly higher than in *C. suis*-negative ones for whole blood from Jiangsu (13.3%, 6/45 vs. 0.0%, 0/40; P<0.01) and rectal swabs from Yunnan and Zhejiang (87.0%, 47/54 vs. 54.7%, 29/53; P<0.01).

### *C. suis ompA* molecular typing

**Fig. 3.**
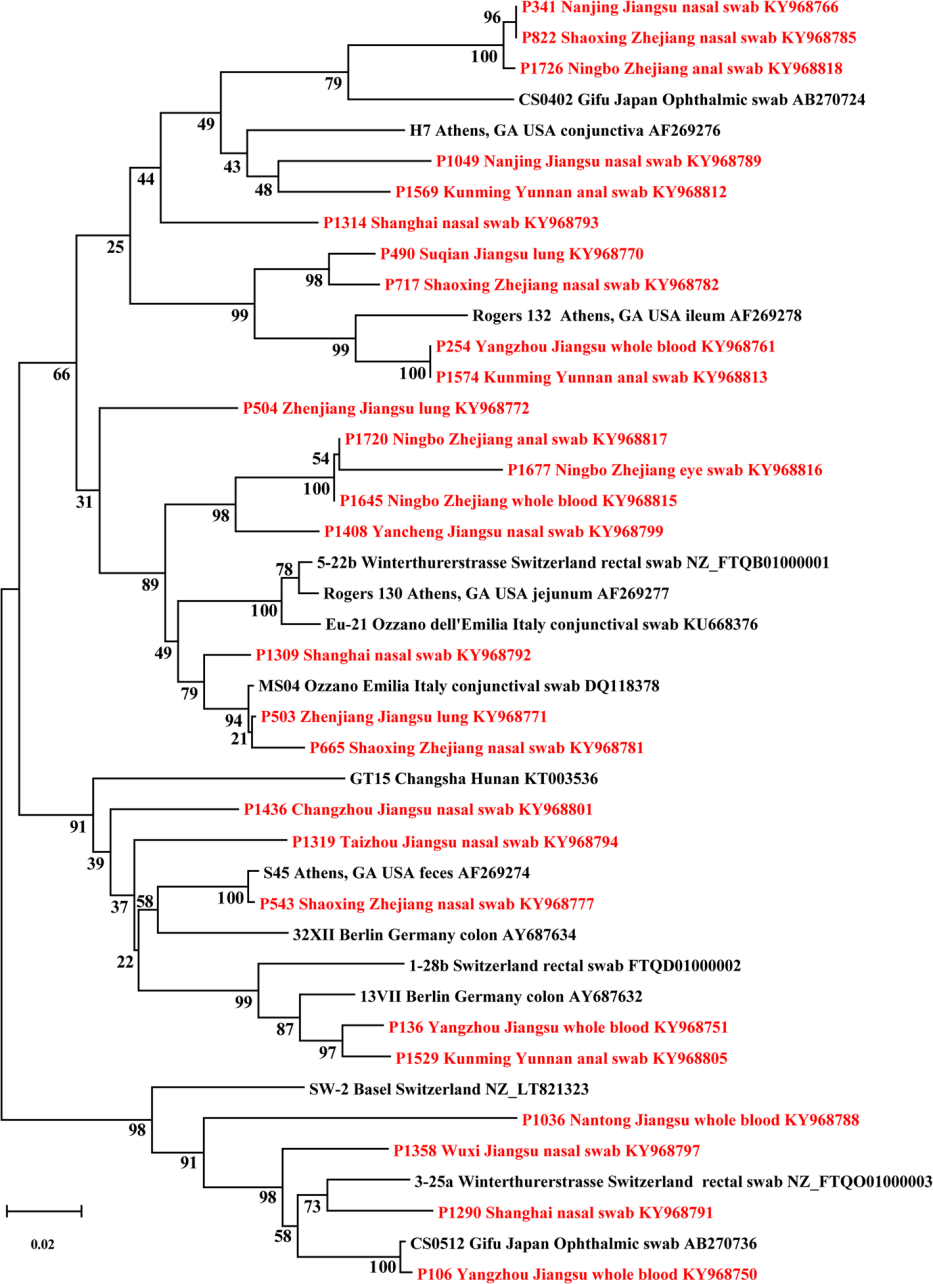
Mid-point rooted NJ phylogenetic tree of the *ompA* variable domains 1-2. A 489-bp fragment encompassing *C. suis ompA* VD1-2 of 27 porcine *C. suis* strains identified in this study (in red font; name of strain, countries, sample type and accession number) are compared with 15 other *C. suis* sequences deposited in GenBank from six countries (Germany, Switzerland, Italy, USA, Japan and China). Branch lengths are measured in nucleotide substitutions and numbers show branching percentages in bootstrap replicates. Scale bar represents the percent sequence diversity.

We obtained 77 distinct partial *ompA* sequences encompassing the variable domain 1 and 2 (VD1-2) from 108 pig *C. suis* strains (Fig. 3, Additional file 1). Phylogenetic analyses showed that the partial *ompA* VD1-2 sequences from *C. suis* strains from China identified in this study are highly polymorphic when compared with the existing *ompA* sequences deposited in GenBank. Percent sequence similarity observed within the Chinese cohort was 76.0%-100%, while between the Chinese cohort and the GenBank sequences, only 64.0%-100% sequence similarity could be observed. When compared with global *ompA* sequences, the Chinese *C. suis ompA* VD1-2 sequences could be resolved into four major clades of the global *ompA* VD1-2 phylogram, suggesting that the global diversity in *C. suis* strains is captured in the Chinese pig population (Figure 3). While considerable sequence variation was observed, some identical strains were also detected including the detection of at least one sequence (Ya/ChPP854/Nasal) in Zhejiang that was identical to the sequence from a *C. suis* strain (AB270743) deposited from Japan. Fine-detailed epidemiological analysis of these sequences further revealed that the same *ompA* sequences could be found in different cities and provinces, suggesting that some strains were more widely distributed than other strains.

### *C. suis* MLST analyses

In this study, we also assessed the genetic diversity of the *C. suis* strains detected using a *C. suis*-specific MLST scheme. The MLST of the 27 global *C. suis* strains confirmed high genetic diversity for this pathogen, identifying 26 novel STs among 27 strains (0.96 ST/strain). Sequence analysis of individual as well as concatenated *C. suis* MLST gene fragments from this study confirmed that the *C. suis* HK genes are conserved and under purifying selection with dN/dS ratios < 1, yet with high haplotype diversity. The number of mutations was comparable between the *C. suis* HK alleles, ranging from 29 SNPs in *gidA* to 12 SNPs in *gatA*, with most resulting mainly in synonymous substitutions (Table 3). Each MLST allele had 3 to 8 predicted putative recombination events.

**Table 3:** Sequence analyses of the 27 *C. suis* MLST alleles and concatenated sequences

Allele	Total number of polymorphisms (Δnt)	No. of non-synonymous substitutions	No. of synonymous substitutions	dN/dS ratio	Number of haplotypes
*gat*A (425 bp)	12	1	11	0.010	11
*opp*A_3 (468 bp)	17	3	14	0.061	12
*hfl*X (435 bp)	24	2	22	0.022	18
*gid*A (474 bp)	29	4	25	0.074	18
*eno*A (381 bp)	23	1	22	0.007	23
*hem*N (432 bp)	17	2	15	0.113	14
*fum*C (465 bp)	14	2	12	0.024	14
Concatenated (3080 bp)	136	15	121	-	26

**Fig. 4.**
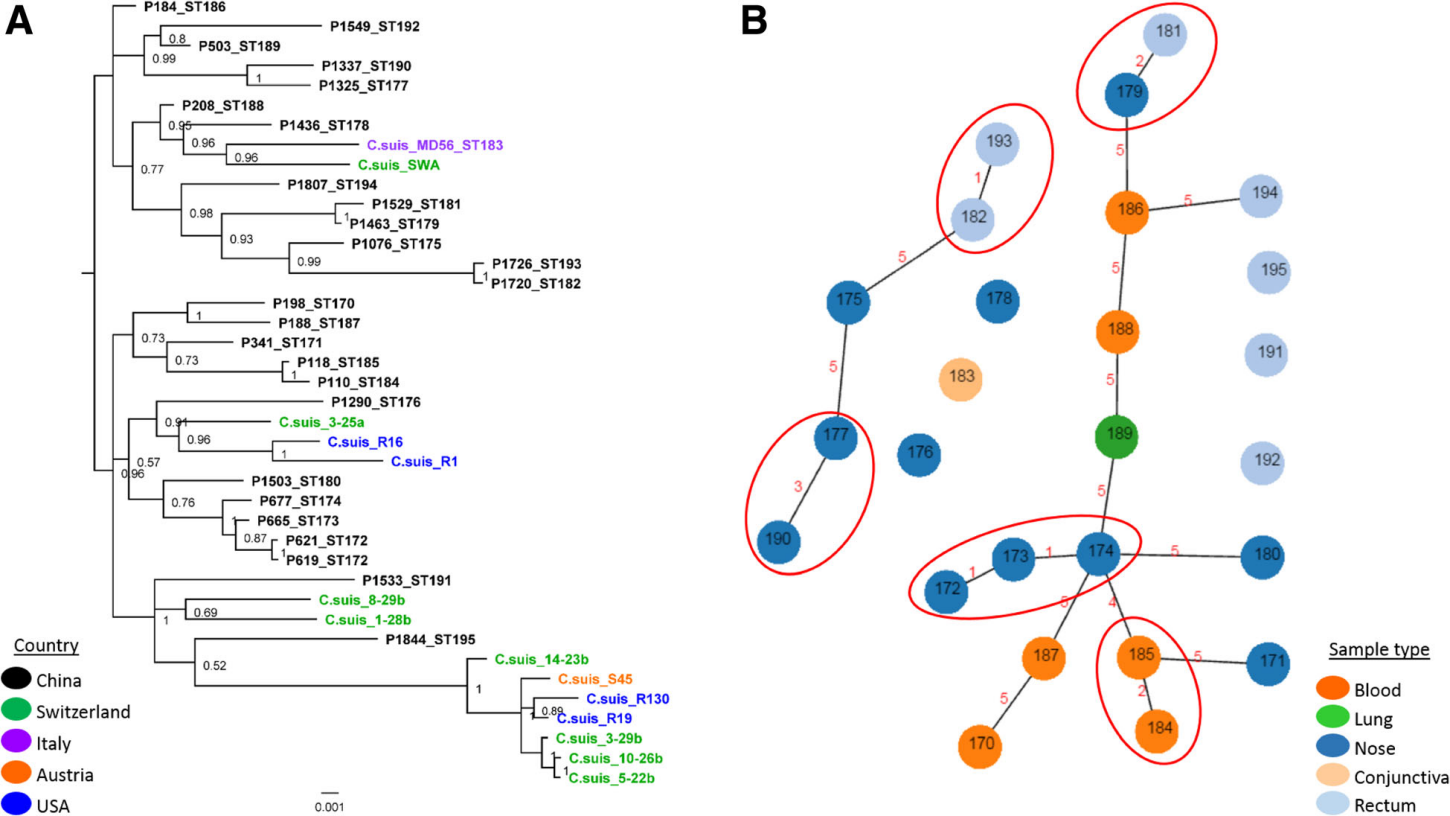
Phylogenetic and cluster analyses of *C. suis* STs. **a**: Bayesian phylogenetic analysis of the concatenated sequences of seven MLST fragments of 40 *C. suis* strains. Posterior probabilities are displayed on tree nodes, while the geographical origin of the strain is indicated by different colors as outlined in the legend; **b**: Cluster analyses of *C. suis* STs, N locus difference is indicated on the link between the nodes. Clonal complexes are circled in red. Sample types from which the *C. suis* STs were derived are indicated by the colors, as outlined in the legend.

Due to its congruency with whole or core genome phylogeny, the concatenated MLST sequences have been previously used to infer phylogenetic relationships between the strains of other chlamydial species [42]. To assess the phylogenetic relationships between the Chinese and other global *C. suis* strains, a mid-point rooted Bayesian phylogenetic tree was constructed from an alignment of a total of 40 strains (Fig. 4a). Using *C. trachomatis* as an out-group, the Chinese isolates resolved into seven diverse larger clades, clustering on their own or with European and US isolates. In their own well-supported clades, Chinese *C. suis* strains formed many distinct lineages. When clustered with European and the USA isolates within a larger clade, Chinese isolates also grouped in separate but diverse sub-clades. Similarly, US and European strains also formed distinct well-supported clades (Fig. 4a).

The phylogenetic analyses further resolved the on-farm epidemiology of Chinese *C. suis* infections including evidence of sub-clades consisting of: a) related STs obtained from various anatomical sites of different hosts (as observed in the sub-clade consisting of nasal STs 190 and 177, rectal ST 192 and lung ST 189; and b) closely related STs obtained from the same anatomical site from different hosts (such as all nasal STs 172, 173, 174 and 180 sub-clade and blood STs 185 and 184, and 170 and 187 sub-clades).

The *C. suis* STs cluster analyses and minimum spanning tree also confirmed high ST diversity (Fig. 4b). Using a relaxed fit where STs must share at least 4/7 alleles, the 26 STs clustered into five clonal complexes (CCs, and 15 satellite STs (Figure 4b). Only two CCs resolved based on a stringent fit differing by a single locus. CC 1 consisted of nasal only isolates denoted STs 172, 173 and 174, with ST 172 as a predicted progenitor, while CC 2 consisted of rectal STs 182 and 193, with ST 182 predicted as a putative progenitor.

### Comparison of *C. suis* MLST and *ompA* phylogenies

**Fig. 5.**
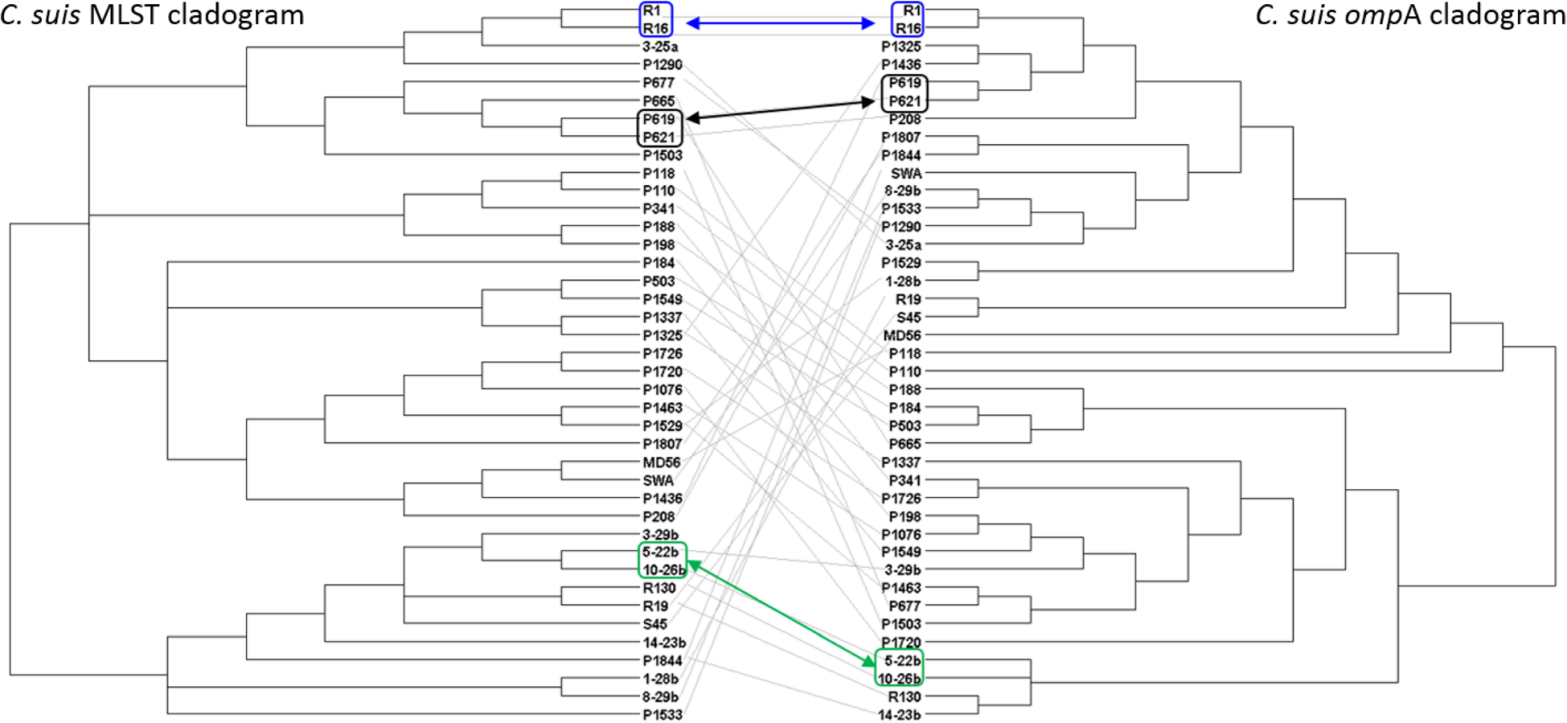
Tanglegram of *C. suis* MLST and *ompA* Bayesian trees, transformed as a cladograms, from this study. Same positioning of the taxa is indicated by colored squares and arrows, where blue indicates the USA *C. suis* isolates, black Chinese and green Swiss isolates.

In order to further compare phylogenetic relationships of *C. suis* strains, we produced a tanglegram of both MLST and *ompA* trees to estimate phylogenetic positioning for each taxa (strain) (Fig. 5). As observed in Fig. 5, only six taxa, including the USA R1 and R16, Chinese P619 and P621; and Swiss 5-22b and 10-26b strains, maintained the same phylogenetic clustering in both trees, whereas all other taxa clustered differently in each tree.

We additionally tested for the recombination break points and changes in topology in the MLST and *ompA* alignments using DualBrothers. Based on observed break-points (Additional files 2, 3 and 4), we can infer at least one putative recombination event in the *C. suis* MLST alignment, and at least four in the *ompA* alignment.

## Discussion

The present study investigated the prevalence of *C. suis* in five different sample types from 2,137 pigs from 19 regions of 10 provinces in China. The present PCR methodology represents a reliable diagnostic tool for rapid, highly sensitive and specific detection of *C. suis*, bypassing the need for *C. suis* cell culture [29, 31, 43]. We reported an overall prevalence of 62.4% (1,334/2,137) positive animals in the genus-specific FRET-PCR with our results confirming a high prevalence of *C. suis* in all pig herds. We failed to detect any evidence of *C. abortus*, *C. pecorum* and *C. psittaci* DNA. This study provides the first evidence for *C. suis* infection in commercially raised pigs in China. Furthermore, this is also the first report to find that 86.1% (93/108) SPF pigs were *C. suis*-positive.

Our study was the first to show the presence of *C. suis* in blood samples. For paired samples from the same host, nasal, conjunctival and rectal swabs were *C. suis*-positive when the whole blood samples were also *C. suis*-positive. Furthermore, 6/23 lung samples were *C. suis*-positive, suggesting that systemic infection is a common outcome of the haemtogenous spread of the bacteria.

In this study, generally, the infection rate of sows was significantly lower when compared with suckling, weaned and finisher pigs (Fig. 2). These observations might be attributed to antibiotic treatment and good housing conditions for sows or protective immunity in the adult animals. Housing conditions and management systems might contribute to controlling the transmission of chlamydial infections in pigs [10]. However, these results show that despite these practices, *C. suis* was prevalent in pigs at all stages of their production cycle.

While *C. suis* was found to be the most common species in pigs [44], this organism was mostly often found in the intestine [45–47] and conjunctiva [48, 49], and in other sites, including the genital tract [50], nasal specimens [51], lung [52] and the aborted fetuses [53].

In terms of transmission, in this study, the prevalence of *C. suis* DNA in rectal swabs was significantly higher when compared to those taken from nose and eyes, with whole blood samples having the lowest *C. suis* DNA incidence. This result is most likely due to the gastrointestinal tract being the primary site infection and chlamydial replication. Alternatively, it is also possible that pigs can swallow *C. suis* eliminated from the respiratory tract by mucociliary clearance [51]. Furthermore, the incidence of *C. suis* DNA in rectal, nasal, conjunctival swab samples and whole blood samples was significantly higher for weaned pigs when compared with sows. Data of the present study may suggest the potential *C. suis* transmission via a fecal-oral route. Besides the fecal-oral route as a potential route of transmission, the transmission may presumably also occur via exchange of body fluids, particularly secretions from the conjunctiva and nose, which may also lead to aerosol formation [10]. Hamonic et al. [54] recommended that it is possible that viable *C suis* shed in boar semen could cross current biosecurity barriers, meaning vertical transmission may also need to be considered a route of transmission.

Since 1998, the number of reports of TcR *C. suis* infections in pigs are growing in the USA, Italy, Cyprus, Germany, Israel, Switzerland and Belgium [18–20, 22, 55]. In the present study, the *tet*(C) gene was detected in clinical samples from 3 provinces, which suggests that the presence of the *tet*(C) gene in *C. suis* is common in the Chinese domestic pig population. Selective pressure from tetracycline as drug may be responsible for recent bottlenecks in *C. suis* populations [26]. In this study, we were able to test only clinical samples instead of *C. suis* isolates. However, we cannot exclude that the *tet*(C) gene of other bacteria was detected, and 54.7% *C. suis*-negative rectal swab samples was found to be *tet*(C) positive in this study. For both whole blood and rectal swabs samples in this study, the *tet*(C) positivity of *C. suis*-positive samples were significantly higher than *C. suis*-negative samples, suggesting these *C. suis* strains contained *tet*(C) gene. This may be associated with the inclusion of 10-50 gram oxytetracycline in every 1,000 kilogram pig feed in the farms examined and in the Chinese pig industry. Given the apparently prevalence of this *tet*(C) positivity in *C. suis*, it is concerning to note that, Suchland et al. [23] previously demonstrated the *in vitro* horizontal transfer of tetracycline resistance from *C. suis* to clinical strains of *C. trachomatis*, an important human pathogen. As such, *C. suis* may transfer tetracycline resistance to other chlamydial species, through mixed infections of livestock or farmers. Therefore, for the Chinese pig industry and public health, effective preventive action, such as probiotics or vaccines, should be promoted to counteract it. Targeted surveillance of tetracycline-resistant *C. suis* strains may also be warranted to curb the spread of these unusual chlamydial strains.

Phylogenetic analyses showed that the *ompA* VD1-2 gene fragment of the *C. suis* strains is highly polymorphic (Fig. 3). *C. suis* MLST confirmed these observations, possibly suggesting a shared ancestry of some Chinese strains with those described in the USA and Europe. Combining phylogenetic and cluster analyses findings, and in absence of typing paired samples from a single host, *C. suis* epidemiology nevertheless appears complex.

The genome of *C. suis* is very plastic combining unprecedented diversity with significant levels of recombination predicted between strains as well as plasmid exchange [26, 56]. Although we only used MLST and a partial *ompA* sequence, phylogenetic and sequence analyses from the present study provided evidence of recombination in Chinese *C. suis* strains. We observed differing phylogenetic positioning of the strains when constructing phylogenies using different genes, and identified of putative change points in tree topology and sequence, all indicative of recombination in the Chinese strains as well. In combination with the extreme prevalence in swine herds, the well-known fusion of chlamydial inclusion in closely related *C. trachomatis* [23], but not other chlamydial lineages, may be the pre-requisite for the unprecedented genome-wide recombination frequency of *C. suis*. If so, these mechanisms would continuously create, even within single farms, new strains that explore the full evolutionary bandwidth and enable *C. suis* escape immune protection of the host. Systematic genome sequencing may reveal if *C. suis* strains of different geographic origin that are identical at, e.g., the *ompA* locus, such as Chinese strain P854 and Japanese strain AB270743, are truly identical or are the result of convergent evolution at this locus, but are otherwise divergent across the rest of the *C. suis* chromosome.

## Conclusions

The epidemiological surveys in this study indicate that *C. suis* infection in Chinese pigs is common. Molecular typing of detected strains suggest that, like elsewhere, *C. suis* are genetically diverse and that the global diversity of this pathogen is reflected in the diversity of strains detected in Chinese pigs. Further research should be performed to study the route of transmission for *C. suis*, and the potential impacts of *C. suis* on pig production.

## Additional files

Additional file 1: Mid-point rooted NJ phylogeny of ompA variable domains 1-2. A 489 bp region encompassing ompA VD1-2 sequences of 77 porcine *C. suis* strains identified in this study (in red font; name of strain, countries, sample type and accession number) are compared with 50 other *C. suis* sequences deposited in GenBank from six countries: Germany, Switzerland, Italy, USA, Japan and China (in black font). Branch lengths are measured in nucleotide substitutions and numbers show branching percentages in bootstrap replicates. Scale bar represents the percent sequence diversity. (PDF 376 kb)

Additional file 2: DualBrother recombination detection using the 489bp *ompA* fragment alignment of 26 Chinese *C. suis* strains. The top plot shows marginal posterior probabilities of the four most probable tree topologies, where break and change points of topologies are indicative of recombination. The last two plots show 95% Bayesian confidence interval (shaded in green) of the Kappa transition/transversion ratio, and average divergence Mu. (PDF 141 kb)

Additional file 3: DualBrother recombination detection using the 489bp ompA fragment alignment of all 40 C. suis strains used in this study. The top plot shows marginal posterior probabilities of the four most probable tree topologies, where break and change points of topologies are indicative of recombination. The last two plots show 95% Bayesian confidence interval (shaded in green) of the Kappa transition/transversion ratio, and average divergence Mu. (PDF 157 kb)

Additional file 4: DualBrother recombination detection using the concatenated 3,080 bp MLST alignment of all 40 *C. suis* strains used in this study. The top plot shows marginal posterior probabilities of the four most probable tree topologies, where break and change points of topologies are indicative of recombination. The last two plots show 95% Bayesian confidence interval (shaded in green) of the Kappa transition/transversion ratio, and average divergence Mu. (PDF 143 kb)

## Abbreviations

FRET: fluorescence resonance energy transfer
MCMC: Markov Chain Monte Carlo
*ompA*: out major protein A
