# Supplementary material for: Asymptomatic infections with highly polymorphic Chlamydia suis are ubiquitous in pigs
Source: BMC Vet Res. 2017 Dec 1;13:370. doi: 10.1186/s12917-017-1295-x (PMC5710075; doi:10.1186/s12917-017-1295-x)
Supplement: Supplementary file 1 — Mid-point rooted NJ phylogeny of ompA variable domains 1-2. A 489 bp region encompassing ompA VD1-2 sequences of 77 porcine C. suis strains identified in this study (in red font; name of strain, countries, sample type and accession number) are compared with 50 other C. suis sequences deposited in GenBank from six countries: Germany, Switzerland, Italy, USA, Japan and China (in black font). Branch lengths are measured in nucleotide substitutions and numbers show branching percentages in bootstrap replicates. Scale bar represents the percent sequence diversity. (PDF 376 kb) [file 12917_2017_1295_MOESM1_ESM.pdf]

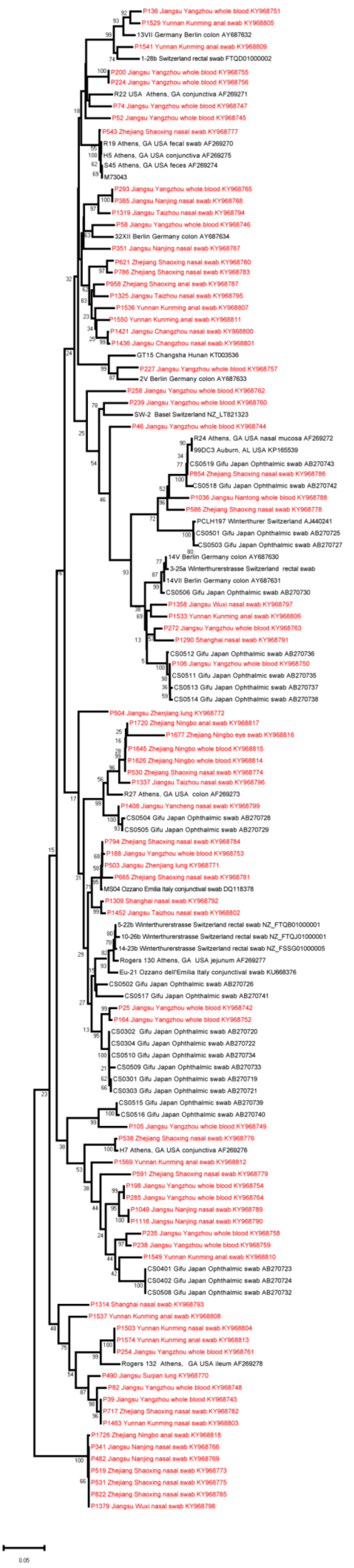
P136 Jiangsu Yangzhou whole blood KY968751
P1529 Yunnan Kunming anal swab KY968805
13VII Germany Berlin colon AY687632
P1541 Yunnan Kunming anal swab KY968809
1-28b Switzerland rectal swab FTQD01000002
P200 Jiangsu Yangzhou whole blood KY968755
P224 Jiangsu Yangzhou whole blood KY968756
R22 USA Athens, GA conjunctiva AF269271
P74 Jiangsu Yangzhou whole blood KY968747
P52 Jiangsu Yangzhou whole blood KY968745
P543 Zhejiang Shaoxing nasal swab KY968777
R19 Athens, GA USA fecal swab AF269270
H5 Athens, GA USA conjunctiva AF269275
S45 Athens, GA USA feces AF269274
M73043
P293 Jiangsu Yangzhou whole blood KY968765
P385 Jiangsu Nanjing nasal swab KY968768
P1319 Jiangsu Taizhou nasal swab KY968794
P58 Jiangsu Yangzhou whole blood KY968746
32XII Berlin Germany colon AY687634
P351 Jiangsu Nanjing nasal swab KY968767
P621 Zhejiang Shaoxing nasal swab KY968780
P786 Zhejiang Shaoxing nasal swab KY968783
P958 Zhejiang Shaoxing anal swab KY968787
P1325 Jiangsu Taizhou nasal swab KY968795
P1536 Yunnan Kunming anal swab KY968807
P1550 Yunnan Kunming anal swab KY968811
P1421 Jiangsu Changzhou nasal swab KY968800
P1436 Jiangsu Changzhou nasal swab KY968801
GT15 Changsha Hunan KT003536
P227 Jiangsu Yangzhou whole blood KY968757
2V Berlin Germany colon AY687633
P258 Jiangsu Yangzhou whole blood KY968762
P239 Jiangsu Yangzhou whole blood KY968760
SW-2 Basel Switzerland NZ_LT821323
P46 Jiangsu Yangzhou whole blood KY968744
R24 Athens, GA USA nasal mucosa AF269272
99DC3 Auburn, AL USA KP165539
CS0519 Gifu Japan Ophthalmic swab AB270743
P854 Zhejiang Shaoxing nasal swab KY968786
CS0518 Gifu Japan Ophthalmic swab AB270742
P1036 Jiangsu Nantong whole blood KY968788
P586 Zhejiang Shaoxing nasal swab KY968778
PCLH197 Winterthurer Switzerland AJ440241
CS0501 Gifu Japan Ophthalmic swab AB270725
CS0503 Gifu Japan Ophthalmic swab AB270727
14V Berlin Germany colon AY687630
3-25a Winterthurerstrasse Switzerland rectal swab
14VII Berlin Germany colon AY687631
CS0506 Gifu Japan Ophthalmic swab AB270730
P1358 Jiangsu Wuxi nasal swab KY968797
P1533 Yunnan Kunming anal swab KY968806
P272 Jiangsu Yangzhou whole blood KY968763
P1290 Shanghai nasal swab KY968791
CS0512 Gifu Japan Ophthalmic swab AB270736
P106 Jiangsu Yangzhou whole blood KY968750
CS0511 Gifu Japan Ophthalmic swab AB270735
CS0513 Gifu Japan Ophthalmic swab AB270737
CS0514 Gifu Japan Ophthalmic swab AB270738
P504 Jiangsu Zhenjiang lung KY968772
P1720 Zhejiang Ningbo anal swab KY968817
P1677 Zhejiang Ningbo eye swab KY968816
P1645 Zhejiang Ningbo whole blood KY968815
P1626 Zhejiang Ningbo whole blood KY968814
P530 Zhejiang Shaoxing nasal swab KY968774
P1337 Jiangsu Taizhou nasal swab KY968796
R27 Athens, GA USA colon AF269273
P1408 Jiangsu Yancheng nasal swab KY968799
CS0504 Gifu Japan Ophthalmic swab AB270728
CS0505 Gifu Japan Ophthalmic swab AB270729
P794 Zhejiang Shaoxing nasal swab KY968784
P188 Jiangsu Yangzhou whole blood KY968753
P503 Jiangsu Zhenjiang lung KY968771
P665 Zhejiang Shaoxing nasal swab KY968781
MS04 Ozzano Emilia Italy conjunctival swab DQ118378
P1309 Shanghai nasal swab KY968792
P1452 Jiangsu Taizhou nasal swab KY968802
5-22b Winterthurerstrasse Switzerland rectal swab NZ_FTQB01000001
10-26b Winterthurerstrasse Switzerland rectal swab NZ_FTQJ01000001
14-23b Winterthurerstrasse Switzerland rectal swab NZ_FSSG01000005
Rogers 130 Athens, GA USA jejunum AF269277
Eu-21 Ozzano dell'Emilia Italy conjunctival swab KU668376
CS0502 Gifu Japan Ophthalmic swab AB270726
CS0517 Gifu Japan Ophthalmic swab AB270741
P25 Jiangsu Yangzhou whole blood KY968742
P164 Jiangsu Yangzhou whole blood KY968752
CS0302 Gifu Japan Ophthalmic swab AB270720
CS0304 Gifu Japan Ophthalmic swab AB270722
CS0510 Gifu Japan Ophthalmic swab AB270734
CS0509 Gifu Japan Ophthalmic swab AB270733
CS0301 Gifu Japan Ophthalmic swab AB270719
CS0303 Gifu Japan Ophthalmic swab AB270721
CS0515 Gifu Japan Ophthalmic swab AB270739
CS0516 Gifu Japan Ophthalmic swab AB270740
P105 Jiangsu Yangzhou whole blood KY968749
P538 Zhejiang Shaoxing nasal swab KY968776
H7 Athens, GA USA conjunctiva AF269276
P1569 Yunnan Kunming anal swab KY968812
P591 Zhejiang Shaoxing nasal swab KY968779
P198 Jiangsu Yangzhou whole blood KY968754
P285 Jiangsu Yangzhou whole blood KY968764
P1049 Jiangsu Nanjing nasal swab KY968789
P1116 Jiangsu Nanjing nasal swab KY968790
P235 Jiangsu Yangzhou whole blood KY968758
P238 Jiangsu Yangzhou whole blood KY968759
P1549 Yunnan Kunming anal swab KY968810
CS0401 Gifu Japan Ophthalmic swab AB270723
CS0402 Gifu Japan Ophthalmic swab AB270724
CS0508 Gifu Japan Ophthalmic swab AB270732
P1314 Shanghai nasal swab KY968793
P1537 Yunnan Kunming anal swab KY968808
P1503 Yunnan Kunming nasal swab KY968804
P1574 Yunnan Kunming anal swab KY968813
P254 Jiangsu Yangzhou whole blood KY968761
Rogers 132 Athens, GA USA ileum AF269278
P490 Jiangsu Suqian lung KY968770
P82 Jiangsu Yangzhou whole blood KY968748
P39 Jiangsu Yangzhou whole blood KY968743
P717 Zhejiang Shaoxing nasal swab KY968782
P1463 Yunnan Kunming nasal swab KY968803
P1726 Zhejiang Ningbo anal swab KY968818
P341 Jiangsu Nanjing nasal swab KY968766
P482 Jiangsu Nanjing nasal swab KY968769
P519 Zhejiang Shaoxing nasal swab KY968773
P531 Zhejiang Shaoxing nasal swab KY968775
P822 Zhejiang Shaoxing nasal swab KY968785
P1379 Jiangsu Wuxi nasal swab KY968798
0.05

1

Supplementary Figure 1. Mid-point rooted NJ phylogeny of *ompA* variable domains 1-2. A 489 bp region encompassing *ompA* VD1-2 sequences of 77 porcine *C. suis* strains identified in this study (in red font; name of strain, countries, sample type and accession number) are compared with 50 other *C. suis* sequences deposited in GenBank from six countries: Germany, Switzerland, Italy, USA, Japan and China (in black font). Branch lengths are measured in nucleotide substitutions and numbers show branching percentages in bootstrap replicates. Scale bar represents the percent sequence diversity.
