# Supplementary material for: Asymptomatic infections with highly polymorphic Chlamydia suis are ubiquitous in pigs
Source: BMC Vet Res. 2017 Dec 1;13:370. doi: 10.1186/s12917-017-1295-x (PMC5710075; doi:10.1186/s12917-017-1295-x)
Supplement: Supplementary file 2 — DualBrother recombination detection using the 489bp ompA fragment alignment of 26 Chinese C. suis strains. The top plot shows marginal posterior probabilities of the four most probable tree topologies, where break and change points of topologies are indicative of recombination. The last two plots show 95% Bayesian confidence interval (shaded in green) of the Kappa transition/transversion ratio, and average divergence Mu. (PDF 141 kb) [file 12917_2017_1295_MOESM2_ESM.pdf]

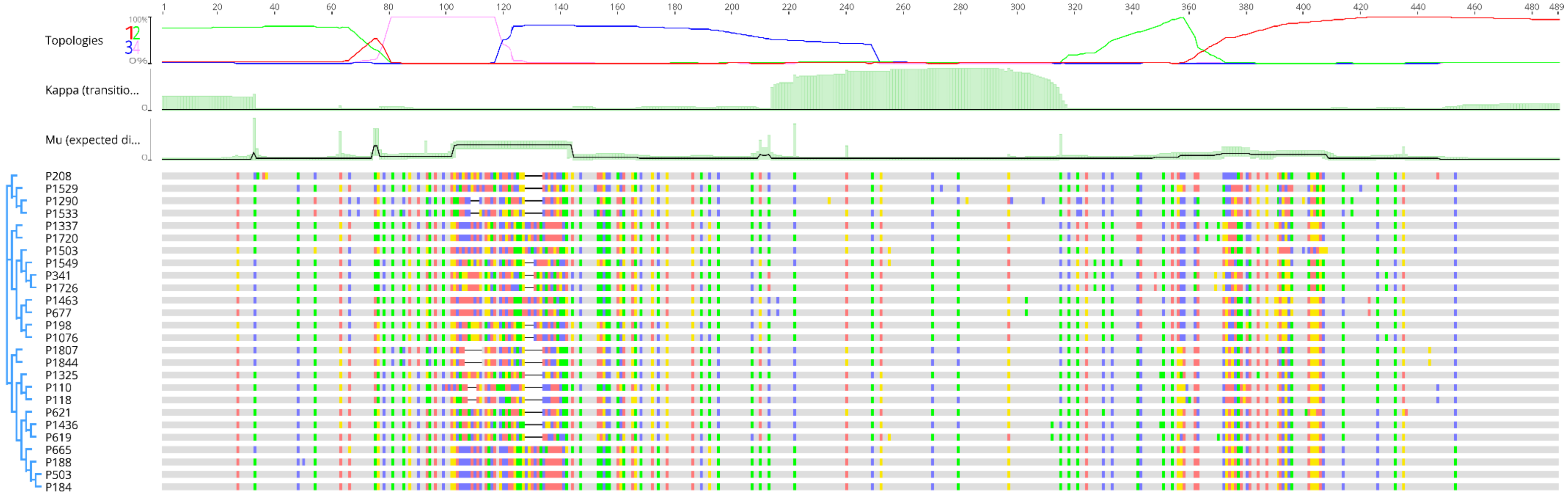

Supplementary Figure 2. DualBrother recombination detection using the 489bp *ompA* fragment alignment of 26 Chinese *C. suis* strains. The top plot shows marginal posterior probabilities of the four most probable tree topologies, where break and change points of topologies are indicative of recombination. The last two plots show 95% Bayesian confidence interval (shaded in green) of the Kappa transition/transversion ratio, and average divergence Mu.
