# Supplementary material for: Asymptomatic infections with highly polymorphic Chlamydia suis are ubiquitous in pigs
Source: BMC Vet Res. 2017 Dec 1;13:370. doi: 10.1186/s12917-017-1295-x (PMC5710075; doi:10.1186/s12917-017-1295-x)
Supplement: Supplementary file 4 — DualBrother recombination detection using the concatenated 3,080 bp MLST alignment of all 40 C. suis strains used in this study. The top plot shows marginal posterior probabilities of the four most probable tree topologies, where break and change points of topologies are indicative of recombination. The last two plots show 95% Bayesian confidence interval (shaded in green) of the Kappa transition/transversion ratio, and average divergence Mu. (PDF 143 kb) [file 12917_2017_1295_MOESM4_ESM.pdf]

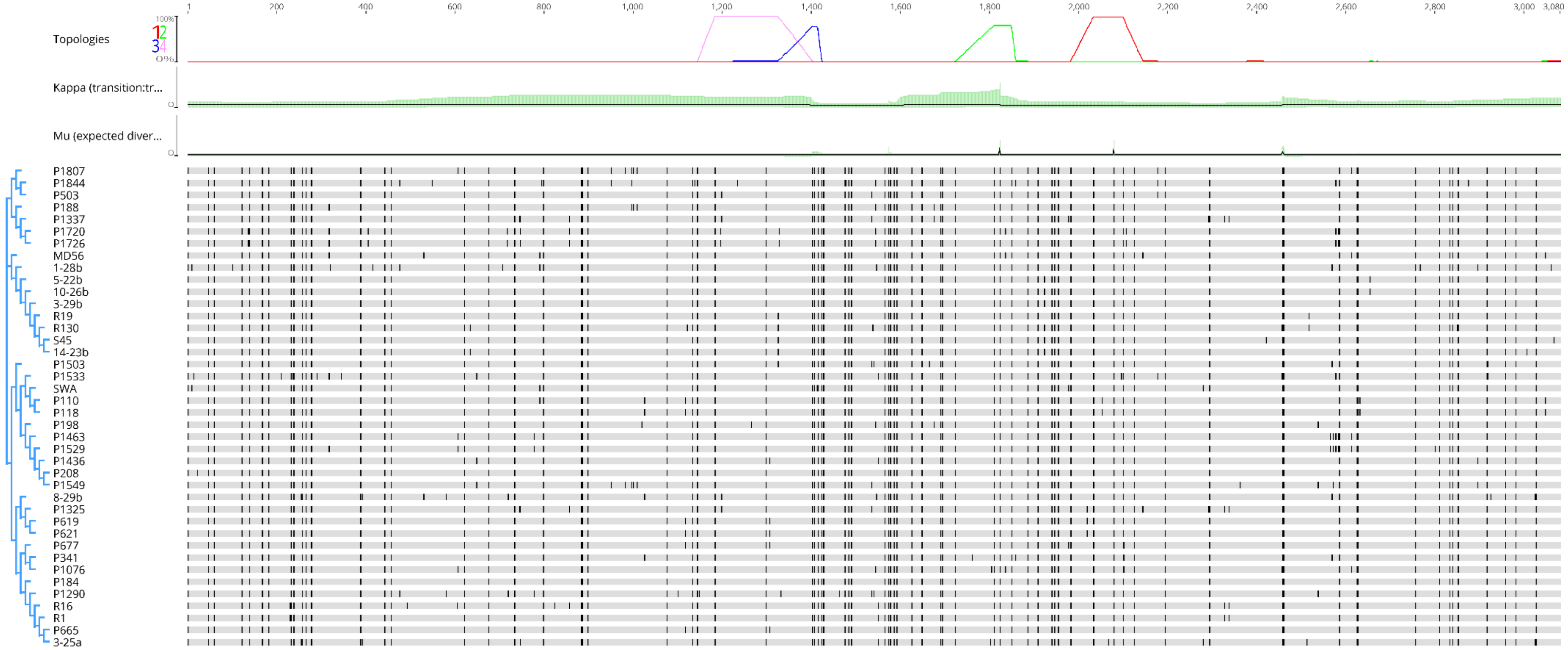

Supplementary Figure 4. DualBrother recombination detection using the concatenated 3,080 bp MLST alignment of all 40 *C. suis* strains used in this study. The plots follow the same definitions as above in Supplementary Figure 2.
